# Efficacy of Wee1 G2 Checkpoint Kinase and Mouse Double Minute 2 Homolog Inhibitors in Gastrointestinal Stromal Tumors Determined by p53 Status

**DOI:** 10.32604/or.2025.066672

**Published:** 2025-10-22

**Authors:** Chiao-Ping Chen, Yan-Jei Tang, You-Yan Cai, Yi-Ru Pan, Chun-Nan Yeh, Wen-Kuan Huang, Chih-Hong Lo, Yu-Tien Hsiao, Hsuan-Jen Shih, Chiao-En Wu

**Affiliations:** 1 Division of Hematology-Oncology, Department of Internal Medicine, Chang Gung Memorial Hospital at Linkou, College of Medicine, Chang Gung University, Taoyuan, 333, Taiwan; 2 Liver Research Center, Chang Gung Memorial Hospital at Linkou, Taoyuan, 333, Taiwan; 3 Department of General Surgery, Chang Gung Memorial Hospital at Linkou, College of Medicine, Chang Gung University, Taoyuan, 333, Taiwan; 4 Institute of Stem Cell and Translational Cancer Research, Chang Gung Memorial Hospital at Linkou, Taoyuan, 333, Taiwan; 5 Division of Hematology-Oncology, Department of Internal Medicine, New Taipei Municipal TuCheng Hospital (Built and Operated by Chang Gung Medical Foundation), New Taipei City, 236, Taiwan

**Keywords:** Tumor protein p53 (p53), Wee1 G2 checkpoint kinase (Wee1), mouse double minute 2 homolog (MDM2), gastrointestinal stromal tumors (GIST), mouse double minute 2 homolog inhibitor (HDM201), adavosertib

## Abstract

**Background:**

KIT proto-oncogene, receptor tyrosine kinase (KIT, CD117) and platelet-derived growth factor-alpha (PDGFRA) are key drivers of gastrointestinal stromal tumors (GIST), but resistance to targeted therapy often arises from tumor protein p53 (p53) alterations and loss of cell cycle control. However, the role of p53 status in GIST therapeutic potential has rarely been studied, so this study aimed to employ both wild-type and mutant p53 GIST models to investigate how p53 dysfunction influences the efficacy of p53 pathway-targeted therapies.

**Methods:**

The efficacy of the mouse double minute 2 homolog (MDM2) inhibitor (HDM201) and the Wee1 G2 checkpoint kinase (Wee1) inhibitor (adavosertib) was confirmed in both p53 wild-type (p53 WT) and p53 mutant (p53 MT) GIST cells. The anti-proliferative effects were assessed using the Cell Counting Kit-8 (CCK-8) assay. Flow cytometry (FACS) and immunoblotting were employed to evaluate apoptosis and the expression of proteins related to drug efficacy. These findings were further validated in a xenograft model.

**Results:**

HDM201 selectively inhibited growth and triggered apoptosis in p53 WT GIST cells, while adavosertib was effective mainly in p53 MT cells. Western blot analysis revealed that HDM201 increased p53 and p21 levels in p53 WT cells, and adavosertib affected Wee1 and phospho-cdc2 expression in both p53 WT and p53 MT cells. In a xenograft mouse model, HDM201 significantly reduced the tumor volume and weight in p53 WT GIST cells, whereas p53 MT tumors showed only a moderate size reduction with adavosertib, without significant changes.

**Conclusions:**

Our results highlight the importance of p53 status in guiding GIST treatment. p53 WT tumors respond to MDM2 inhibitors, while p53 MT tumors show greater sensitivity to Wee1 inhibitors, supporting p53 pathway targeting as a promising strategy for GIST patients.

## 1 Introduction

Gastrointestinal stromal tumors (GISTs) belong to a category of mesenchymal tumors that originate in the gastrointestinal tract. More than 90% of GISTs overexpress the KIT proto-oncogene, receptor tyrosine kinase (KIT, CD117) protein [1–3]. A deeper understanding of GISTs has emerged with the identification of KIT expression and the *KIT* gene (*c-Kit*) [2,3]. An additional 5%–10% of GISTs contain mutations that overexpress platelet-derived growth factor-alpha (PDGFRA) [4]. Small-molecule targeted therapies utilizing tyrosine kinase inhibitors (TKIs) have been developed to treat GIST [5,6]. While the majority of GISTs are characterized by mutations in the *KIT* or *PDGFRA* kinase genes, approximately 5%–10% of GISTs deviate from this pattern and lack mutations in either *KIT* or *PDGFRA*. These tumors were categorized as KIT/PDGFRA wild-type (WT) GISTs.

The *tumor protein p53* (*TP53*) gene, which encodes the p53 tumor suppressor protein, is often referred to as the guardian of the genome. It is mutated in most human cancers, with mutation frequencies that vary according to the specific cancer type [7]. Wild-type p53 (p53 WT) protein plays a crucial role in the cellular response to DNA damage by initiating cell cycle arrest, DNA repair, and apoptosis [7]. The occurrence and potential prognostic relevance of *TP53* mutations (p53 MT) have been explored in a spectrum of cancer types [7–9]. However, in gastrointestinal stromal tumors (GISTs), p53 has received less attention, as GIST oncogenesis is primarily driven by the dysregulation of KIT and PDGFRA [3]. Key molecular events, such as cyclin-dependent kinase inhibitor 2A (CDKN2A) loss, mouse double minute 2 homolog (MDM2) overexpression, and p53 inactivation, are critical in GIST progression [10]. Given its role in cell cycle regulation and DNA damage response, p53 is implicated in the progression of high-risk GISTs. Although *TP53* mutations are uncommon in GIST, they are more prevalent in high-risk cases and are significantly associated with poorer relapse-free survival [10]. Furthermore, p53 expression serves as an independent prognostic factor in advanced GISTs treated with imatinib [11]. Collectively, these findings highlight the critical role of p53 expression and *TP53* mutations in GIST progression. In p53 WT high-risk GISTs, the p53 signaling pathway is often disrupted due to MDM2 overexpression, which impairs p53’s tumor-suppressive functions, leading to genomic instability, uncontrolled proliferation, and oncogene activation. Consequently, targeting MDM2 or other components of the p53 pathway represents a promising therapeutic approach for GISTs [12,13]. These insights support the development of p53 pathway-targeted therapies as a novel strategy for GIST treatment.

Previous studies have revealed that inhibiting Wee1 G2 checkpoint kinase (Wee1) promotes autophagic degradation of KIT, suggesting that targeting Wee1 could offer a novel therapeutic strategy for GIST [14]. Wee1 plays a critical role in regulating the G2/M cell cycle checkpoint, allowing cancer cells with DNA damage to continue through the replication cycle [14,15]. Furthermore, Wee1 inhibition with adavosertib (MK1775) disrupts the G2/M checkpoint by preventing phosphorylation of cyclin-dependent kinase-1 (CDK1, cdc2), thereby inducing apoptosis in p53-mutant ovarian and lung cancer cells [16,17].

p53 transcriptionally activates MDM2, which in turn promotes negative autoregulation of p53 through ubiquitination [7,18]. As a result, MDM2 is classified as an oncogene, and its amplification or overexpression has the potential to enhance tumor cell proliferation by suppressing the activity of p53 [19]. As MDM2 expression is associated with poor prognosis in GIST, MDM2 inhibitors (nutlin-3) have been shown to suppress growth and induce apoptosis in p53 WT GIST cells [20,21]. HDM201 (siremadlin) is a novel, highly potent, and selective inhibitor of the p53-MDM2 interaction. The use of MDM2 inhibitors may provide an additional strategy beyond targeted therapy drugs such as imatinib, sunitinib, regorafenib, and ripretinib in future studies [22]. This study aimed to confirm the association among p53 status, HDM201, and adavosertib activity in GIST.

## 2 Materials and Methods

### 2.1 Cell Lines

This study used three human GIST cell lines: GIST430 with wild-type p53 (p53 WT), GIST882, and GIST-T1 with mutant p53 (p53 MT). All cell lines were kindly provided by Dr. Nai Jung Chiang (Taipei Veterans General Hospital, Taipei, Taiwan). GIST430 cells were cultured in Iscove’s Modified Dulbecco’s Medium (IMDM) (Gibco, 12440053, Waltham, MA, USA) supplemented with 20% heat-inactivated fetal bovine serum (FBS) (Gibco, 10437-028). GIST882 cells were cultured in Roswell Park Memorial Institute (RPMI) medium 1640 (Gibco, 11875-085) supplemented with 20% FBS, and GIST-T1 cells were maintained in Dulbecco’s Modified Eagle’s Medium (DMEM) (Gibco, 11965-984) supplemented with 10% FBS. All three GIST cell lines were cultured with 100 μg/mL streptomycin and 100 μg/mL penicillin (Gibco, 15140-122) in a humidified atmosphere containing 5% CO_2_ at 37°C. All the cell lines used in this study were verified to be free of mycoplasma contamination and authenticated through short tandem repeat (STR) profiling.

### 2.2 Growth Inhibition Assay

GIST430, GIST882, and GIST-T1 cells were seeded in 96-well plates (3 × 10^3^/well) and incubated overnight, and then treated with HDM201 (obtained from Novartis under a material transfer agreement (MTA), GST0000026313, Basel, Basel-Stadt, Switzerland) or adavosertib (MedChemExpress, Basel, Basel-Stadt, Switzerland) for 96 h. The gradient concentrations of HDM201 from 0–10 μM and adavosertib from 0–5 μM used in this study were determined based on prior *in vitro* studies involving cancer models such as GIST, colon cancer, and ovarian cancer [21,23,24]. Cell viability was assessed using the Cell Counting Kit-8 (CCK8, Dojindo Molecular Technologies, Rockville, MD, USA) method, and the optical density was measured at 450 nm using a microplate reader (Synergy HTX Multi-Mode Reader, BioTek, Winooski, VT, USA).

### 2.3 RNA Interference of TP53

*TP53* siRNA (siTP53-1 sense: 5^′^-CCACCAUCCACUACAACUAdTdT-3^′^, antisense: 5^′^-UAGUUGUA GUGGAUGGUGGdTdT-3^′^ and siTP53-2 sense: 5^′^-GAUGUUCCGAGAGCUGAAUdTdT-3^′^, antisense: 5^′^-AUUCAGCUCUCGGAACAUCdTdT-3^′^) or negative control siRNA (siNC) (sense: 5^′^-UUCU CCGAACGUGUCACGUTT-3^′^, antisense: 5^′^-ACGUGACACGUUCGGAGAATT-3^′^) were transfected into GIST430 cells using DharmaFECT^TM^ transfection reagent (horizon^TM^, Cambridge, UK) for 24 h. Western blotting was performed to confirm knockdown efficacy. After successful *TP53* knockdown, CCK8, a growth inhibition assay, was performed to evaluate the effect of HDM201 or adavosertib on p53 knockdown GIST430 cells.

### 2.4 Western Blotting

GIST cells, treated with siRNA, HDM201, or adavosertib, were lysed in Pierce^TM^ RIPA Lysis and Extraction Buffer (Thermo Scientific, Waltham, MA, USA) supplemented with protease inhibitors (Roche, Basel, Switzerland). The lysates were centrifuged at 12,000× *g* for 20 min at 4°C, and the protein concentration of the supernatants was measured using the Pierce^TM^ BCA Protein Assay Kit (Thermo Scientific, 23225). Total cell lysates were separated on 10% SDS-PAGE gels and transferred to nitrocellulose membranes (Amersham^TM^, Cytiva, Marlborough, MA, USA). Membranes were blocked with 5% skim milk in Tris-buffered saline containing 0.1% Tween 20 (TBST) at room temperature. After blocking, the membranes were incubated overnight at 4°C with primary antibodies, followed by TBST washes and incubation with secondary antibodies, in 5% skim milk for 1–2 h at room temperature. Protein detection was performed using chemiluminescence with horseradish peroxidase-conjugated goat anti-mouse or anti-rabbit secondary antibodies (1:5000, Jackson ImmunoResearch Laboratories, mouse: #115-035-0031, rabbit: #11-035-003, West Grove, PA, USA), and protein bands were visualized using the UVP ChemStudio PLUS Touch imager (Analytik Jena AG, Jena, Germany). The primary antibodies used were as follows: p53 (1:1000, GeneTex, GTX34938, Irvine, CA, USA), KIT (1:1000, ABclonal Technology, A0357, Woburn, MA, USA), phospho-KIT (1:1000, ABclonal Technology, AP0385), Wee1 (1:1000, GeneTex, GTX111392), phospho-Wee1 (1:1000, Cell Signaling Technology, #4910, Danvers, MA, USA), cdc2 (1:1000, GeneTex, GTX108120), phospho-cdc2 (1:1000, GeneTex, GTX128155), MDM2 (1:1000, MilliporeSigma, #MABE281, Burlington, MA, USA), p21 (1:1000, Cell Signaling Technology, #2947) and GAPDH (1:20000, GeneTex, GTX627408).

### 2.5 Fluorescence-Activated Cell Sorting (FACS)

GIST cells in 6-well plates (4 × 10^5^/well) were treated with 1 μM HDM201 or adavosertib for 48 h. For the cell cycle distribution assay, both floating and adherent cells were collected, fixed with cold 70% ethanol, and incubated with Propidium Iodide (PI)/RNase Staining Solution (Cell Signaling Technology, #4087) for 20 min in the dark at room temperature. The samples were then analyzed for DNA content using a FACSCalibur^TM^ flow cytometer (Becton Dickinson, Franklin Lakes, NJ, USA) and CellQuest Pro software (Becton Dickinson), with results processed in FlowJo vX software (Becton Dickinson). The FITC Annexin V Apoptosis Detection Kit I (Becton Dickinson, 556547) was used for the apoptosis assays. The cells were washed twice with cold 1 × PBS (pH 7.4), resuspended in binding buffer, and stained with fluorescein isothiocyanate (FITC), Annexin V, and PI according to manufacturer’s protocol. After 15 min of incubation at room temperature in the dark, the cells were analyzed using a FACSCalibur flow cytometer (Becton Dickinson). The proportion of early and late apoptotic cells was calculated using FlowJo vX software (Becton Dickinson).

### 2.6 Caspase 3/7 Activity Assay

GIST430, GIST882, and GIST-T1 cells were seeded in white 96-well white plates (1 × 10^4^/well) and treated with 1 μ M HDM201 or adavosertib for 24 h. Caspase-3/7 enzymatic activities were measured using a luminometer (Synergy HTX Multi-Mode Reader, BioTek), after adding a 1:1 ratio of Caspase Glo-3/7 reagent (Promega, G8090, Fitchburg, WI, USA) to growth media and incubating for 60 min.

### 2.7 Xenograft Tumorigenicity

The animal studies were approved by the Institutional Animal Care and Use Committee (IACUC, No. 2019032003) of Chang Gung Memorial Hospital at Linkou. All animal experiments were conducted in accordance with the Guide for the Care and Use of Laboratory Animals [25]. The animals were housed in an Association for Assessment and Accreditation of Laboratory Animal Care International (AAALAC)-approved facility in our hospital, under controlled temperature (24°C) and a 12-h light/dark cycle.

A total of 20 specific pathogen-free, immunodeficient NonObese Diabetic/Severe Combined Immunodeficiency (NOD/SCID) male mice, aged four weeks and weighing about 20 g, were obtained from BioLASCO Co., Ltd., Taiwan. Based on previous studies [26–28], 1 × 10^7^ GIST430 or GIST882 cells were suspended in 50 μL of PBS (pH 7.2) mixed with an equal volume of Matrigel (1:1, Corning, 354262, Corning, NY, USA) and then subcutaneously injected into NOD/SCID mice. When the average tumor size reached 100 mm^3^, the mice were randomized into two groups (five per group) and treated via oral gavage. The GIST430 group received either vehicle (0.5% methylcellulose) or HDM201 (100 mg/kg/day, 2 days/week) according to a previous study [29]. Similarly, the GIST882 group received either vehicle or adavosertib (50 mg/kg/day, 5 days/week) based on a prior study [30]. During the treatment period, tumor size, $\text{volume }\left(\frac{\text{length }\left(\text{L}\right)\times \text{width }{\left(\text{W}\right)}^{2}}{2}\right)$, and pain/distress classifications were monitored twice a week until the largest tumor approached but did not exceed 2 cm in diameter, which was designated as the endpoint. On day 11 in our two animal studies, mice were sacrificed for tumor collection.

### 2.8 Statistical Analysis

**Figure 1:**
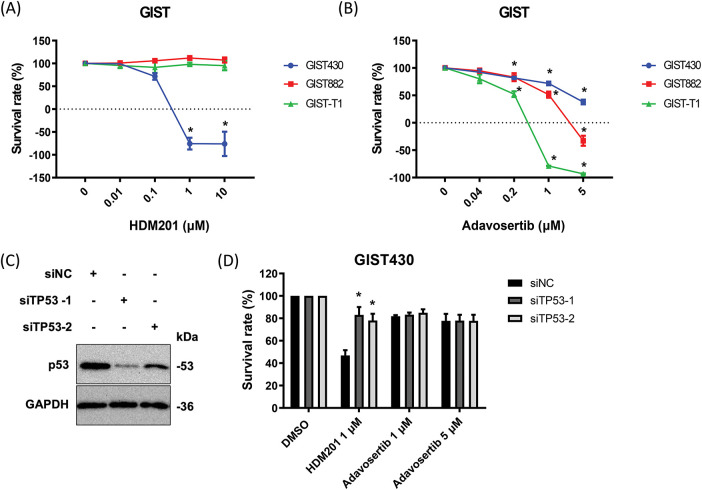
HDM201 and adavosertib enhanced cytotoxicity in gastrointestinal stromal tumors (GIST) cells. **(A)** The survival rate after 96 h of HDM201 treatment, from 0 to 10 μM, in p53 WT cells (GIST430) and p53 MT cells (GIST882 and GIST-T1). **(B)** The survival rate after 96 h of adavosertib treatment, from 0 to 5 μM, in the same three GIST cell lines. **(C)** The knockdown efficiency of *TP53* siRNA (siTP53-1 and siTP53-2) following 48 h of treatment in GIST430 cells. **(D)** The inhibition of cell growth by 1 μM HDM201 was rescued in GIST430 cells upon tumor protein p53 (*TP53*) knockdown compared to the negative control siRNA (siNC) group. The *p* values (mean ± SEM) presented are from three independent experiments. Data were analyzed by two-way ANOVA with Tukey’s post-hoc correction. *p < 0.05

**Figure 2:**
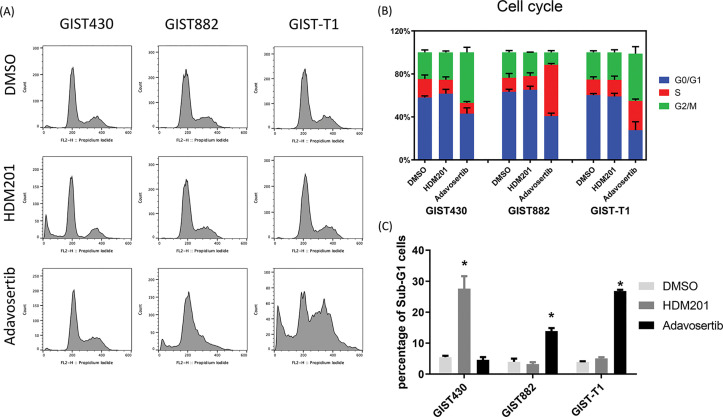
HDM201 and adavosertib increased the proportion of sub-G1 cells in GIST cell lines. (**A, B**) Flow cytometry analysis of cell cycle distribution after 48 h of treatment with 1 μM HDM201 or 1 μM adavosertib. (**C**) Statistical analysis showed that inhibition of mouse double minute 2 homolog (MDM2) or Wee1 G2 checkpoint kinase (Wee1) led to a significant increase in sub-G1 cells in GIST430, GIST882, and GIST-T1 cell lines, respectively. The *p* values (mean ± SEM) presented are from three independent experiments. *p < 0.05

**Figure 3:**
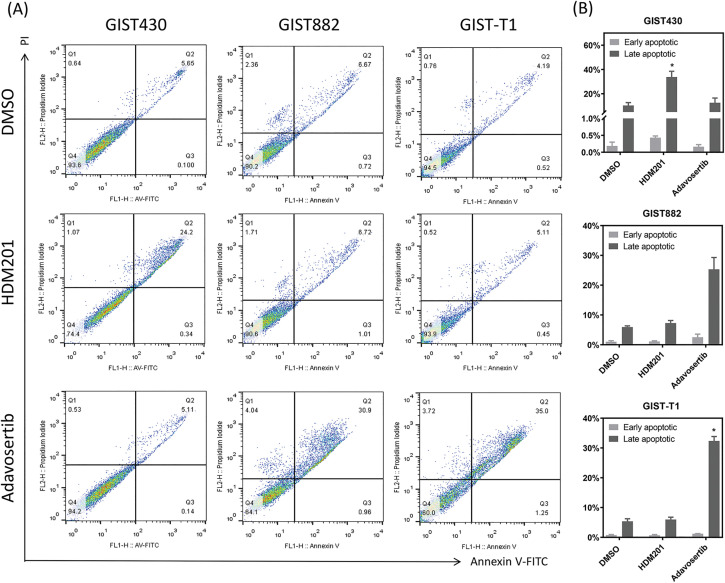

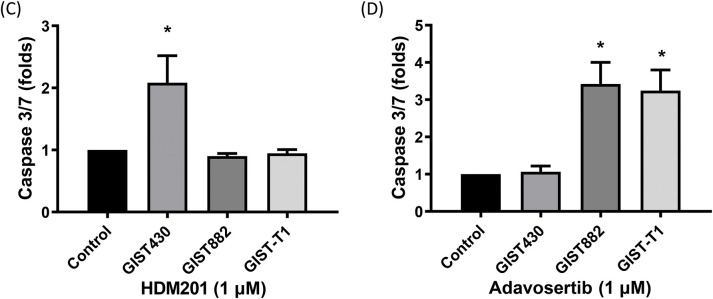
HDM201 and adavosertib enhanced apoptosis in GIST cell lines. (**A, B**) Annexin V and propidium iodide (PI) staining reveal an increase in early and late apoptotic cells following 48 h of treatment with 1 μM HDM201 or 1 μM adavosertib. Caspase 3/7 activity analysis revealed a significant increase in apoptosis after treatment with HDM201 (**C**) or adavosertib (**D**), respectively. The bar graphs show the mean ± SEM from three independent experiments. *p < 0.05

The results shown in Figs. 1–3 represent data from three independent experiments and were analyzed statistically. Values are expressed as the mean ± standard error of the mean (SEM). All statistical tests were performed using the GraphPad Prism 8 software (GraphPad Software, San Diego, CA, USA). *p*-values represent the results of unpaired *t*-tests or two-way analysis of variance (ANOVA), and differences with *p*-values less than 0.05 were considered statistically significant.

## 3 Results

### 3.1 HDM201 and Adavosertib Inhibit GIST Cell Growth in a p53-Dependent Manner

GIST430, GIST882, and GIST-T1 cells were treated with HDM201 (Fig. 1A) and adavosertib (Fig. 1B) for 96 h. Growth inhibition by HDM201 was observed in GIST430 cells, a wild-type p53 cell line, but not in p53 mutated cells, including GIST882 and GIST-T1 cells (Fig. 1A). In contrast, adavosertib inhibited the growth of GIST882 and GIST-T1 cells but not of GIST430 cells (Fig. 1B). Two siRNAs targeting *TP53* (siTP53-1 and 2) successfully suppressed the expression of p53 in GIST430 cells (Fig. 1C, the uncropped Western blot images are provided in Fig. S1). When GIST430 cells were treated with 1 µM HDM201, the growth inhibitory activity decreased after suppression of wild-type p53 expression indicating that the activity of HDM201 is p53-dependent. However, no significant difference was observed in cell survival among the cells treated with adavosertib after p53 knockdown (Fig. 1D). The cell proliferation assay showed that HDM201 effectively inhibited the growth of p53 WT cells, whereas adavosertib effectively inhibited the growth of p53 MT cells.

### 3.2 HDM201 and Adavosertib Cause Cell Cycle Arrest and Apoptosis in GIST, Depending on p53 Status

GIST cells were treated with HDM201 (1 µM) or adavosertib (1 µM) for 48 h. In GIST430 cells, HDM201 decreased the number of S-phase cells, whereas no significant effect was observed in GIST882 and GIST-T1 cells. Adavosertib treatment led to increased S and G2/M phase arrest in GIST-T1 cells and induced S phase arrest in GIST882 cells (Fig. 2A,B). HDM201 significantly increased the sub-G1 population of GIST430 cells but did not affect GIST882 or GIST-T1 cells. Conversely, adavosertib significantly increased the number of sub-G1 GIST882 and GIST-T1 cells, but not the number of GIST430 cells (Fig. 2A,C). Flow cytometry staining with annexin V showed that HDM201 treatment enhanced apoptosis in GIST430 cells, and adavosertib promoted early and late apoptotic cell production in GIST882 and GIST-T1 cells (Fig. 3A,B). Furthermore, caspase 3/7 activity was increased two-fold in GIST430 cells treated with HDM201 (1 µM) compared to the control group, as observed in GIST882 and GIST-T1 cells (Fig. 3C). When GIST cells were treated with adavosertib, caspase 3/7 activity tripled in GIST882 and GIST-T1 cells compared to that in both control and GIST430 cells (Fig. 3D). Cell cycle distribution, annexin V, and caspase 3/7 results showed that HDM201 decreased the S phase population and increased the sub-G1 phase population and apoptosis in p53 WT cells, whereas adavosertib increased apoptosis in p53 MT cells, along with an increase in the sub-G1 phase and induction of S phase and/or G2/M arrest.

### 3.3 Cell Cycle-Related Protein Activation by HDM201 and Adavosertib

**Figure 4:**
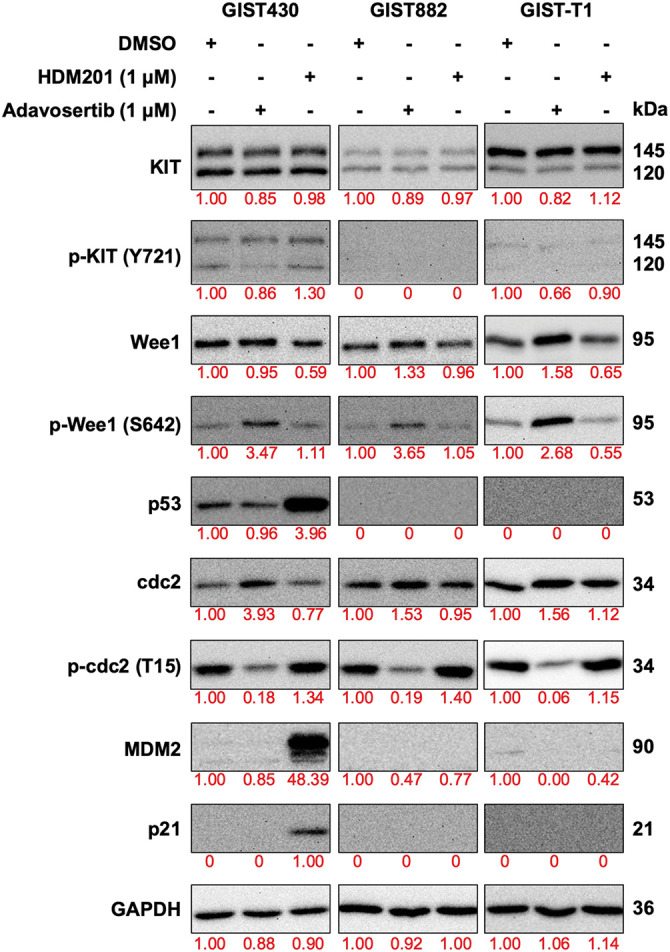
Expression of downstream signaling proteins of MDM2 and Wee1 was analyzed by western blotting after treatment with 1 μM HDM201 or 1 μM adavosertib for 48 h. GAPDH was used as the loading control

To explore the on-target effects of HDM201 and adavosertib, western blot analysis of the three cell lines treated with these drugs was conducted. We analyzed the proteins associated with p53 activity (p53 and p21), which are regulated by MDM2, and cell cycle-related proteins (cdc2, p-cdc2, Wee1, and p-Wee1), which are influenced by adavosertib in these cells (Fig. 4). As depicted by western blotting, compared with DMSO-treated GIST cells, all GIST cells treated with adavosertib increased the expression of phosphorylation of Wee1 and decreased the expression of phospho-cdc2. In addition, no changes were observed in the expression of Wee1 and cdc2. GIST430 treated with HDM201 showed over-expression of p53 and p21; however, no difference was observed in expression between GIST882 and GIST-T1 treated with HDM201 and DMSO. Additionally, consistent with previous studies, the use of adavosertib alone did not affect KIT phosphorylation [31]. Wee1 inhibition did not significantly decrease the phosphorylation of KIT (Y721) in Fig. 4 (The uncropped Western blot images are provided in Figs. S2 and S3). HDM201 reactivated p53 WT but did not influence KIT expression. Western blotting analysis revealed that HDM201 treatment elevated p53 and p21 levels in p53 WT cells, whereas adavosertib modulated Wee1 and phospho-cdc2 expression in both p53 WT and p53 MT cells.

### 3.4 Tumor Responses to HDM201 and Adavosertib in a Xenograft Model

**Figure 5:**
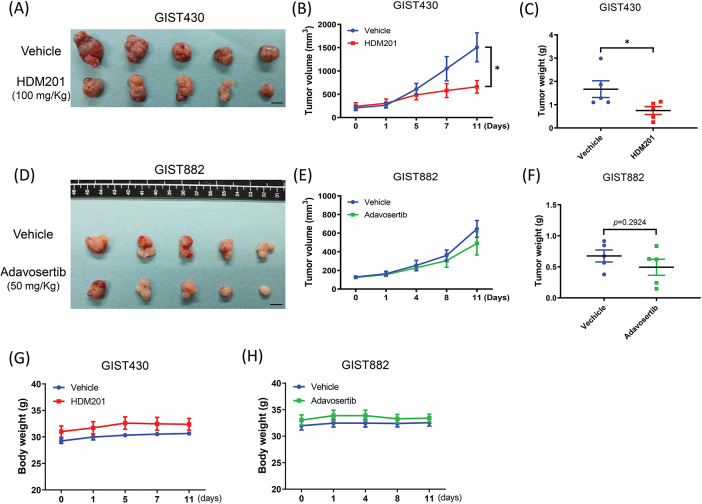
MDM2 inhibition suppressed GIST430 tumor formation. Harvested tumors treated with 100 mg/kg HDM201 in GIST430 (**A**) are displayed. (**B**) Tumor volume increased in the vehicle group but not in the HDM201-treated group. (**C**) Tumor weights were significantly reduced in the HDM201-treated GIST430 xenograft group. Harvested tumors treated with 50 mg/kg adavosertib in GIST882 (**D**) are shown. Wee1 inhibition showed moderate effects on tumor volume (**E**) or weight (**F**) in the GIST882 group. No significant changes in body weight were observed among xenografted mice treated with vehicle, HDM201, or adavosertib in both GIST430 (**G**) and GIST882 (**H**) models. Data are expressed as mean ± SEM. *p < 0.05. Scale bar: 1 cm

GIST430 and GIST882 cells were inoculated subcutaneously into NOD/SCID mice and treated with HDM201 (100 mg/kg, two times per week) or adavosertib (50 mg/kg, five times per week). Compared to the control group, GIST430 tumor volume and weight were significantly decreased in the HDM201 group (Fig. 5A–C). Moreover, GIST882 tumors exhibited a size reduction, although no significant difference was observed in tumor volume and weight between the adavosertib and control groups (Fig. 5D–F). HDM201 treatment led to a significant reduction in the tumor volume and weight of p53 WT GIST cells. In contrast, p53 MT tumors exhibited a moderate decrease in size after adavosertib treatment. Body weight was monitored throughout the treatment period, and no significant changes were observed (Fig. 5G,H).

## 4 Discussion

This study investigated the therapeutic potential of MDM2 and Wee1 inhibitors (HDM201 and adavosertib, respectively) in GIST, with a focus on their p53 dependency. The p53 tumor suppressor protein is mutated in the majority of human cancers, with mutation rates varying according to cancer type [7]. When the *TP53* gene undergoes mutation, it can either lose its normal tumor-suppressive function or gain new oncogenic properties. Consequently, mutant p53 cell lines offer a more faithful representation of actual tumor behavior in cancer research. Certain p53 mutations, such as R175H, R248Q, and R273H, increase cancer cell aggressiveness, promote rapid proliferation, and may even alter cellular metabolism [32,33]. As a result, mutant p53 cell models are pivotal for investigating the oncogenic role of p53 and for developing novel therapies aimed at targeting these mutant variants [34–36].

The GIST cell lines GIST48, GIST430, GIST882, and GIST-T1 are widely used as experimental models for GIST, thanks to their well-characterized mutation sites in the *KIT* gene [37,38]. Among these four lines, GIST882 and GIST-T1 harbor mutant p53, featuring deletions in p53 exon 1 and exons 2–7, respectively [21]. This study maintains GIST430 (p53 WT), GIST882, and GIST-T1 (p53 MT), which can serve as experimental models in GIST preclinical testing for developing potential therapeutics targeting p53 or related pathways. All three cell lines carry KIT mutations. GIST430 carries a primary KIT exon 11 mutation (p.V560_L576del) and a secondary resistance mutation (p.V654A), making it imatinib-resistant. GIST882 harbors a KIT exon 13 mutation (p.K642E), and GIST-T1 has a KIT exon 11 mutation (p.V560_Y578del); both are considered imatinib-sensitive [39]. GIST430 was derived from a GIST that progressed during imatinib mesylate (IM) therapy, thereby conferring IM resistance [40]. GIST882 was established from an untreated human GIST and remains sensitive to IM [41]. GIST-T1, also from an untreated human, is highly sensitive to IM at low nanomolar doses [42]. Although IM exhibits strong anti-proliferative effects, it often fails to induce sufficient apoptosis, resulting in low pathologic complete remission rates and a high rate of secondary progression in metastatic settings [43,44]. These genetic backgrounds reflect clinically relevant KIT mutation subtypes and provide a representative spectrum for investigating therapeutic responses in GIST models. Consequently, more effective treatment is needed. While the overall incidence of *TP53* mutations in GIST is relatively low (3.5%), they are more commonly observed in gastric GISTs, which are associated with poorer recurrence-free survival (RFS) [10]. Accordingly, this study employed both wild-type and mutant p53 GIST models to examine how p53 dysfunction influences GIST biology and to assess its effect on the efficacy of p53-targeted therapies, laying the groundwork for future clinical research.

**Figure 6:**
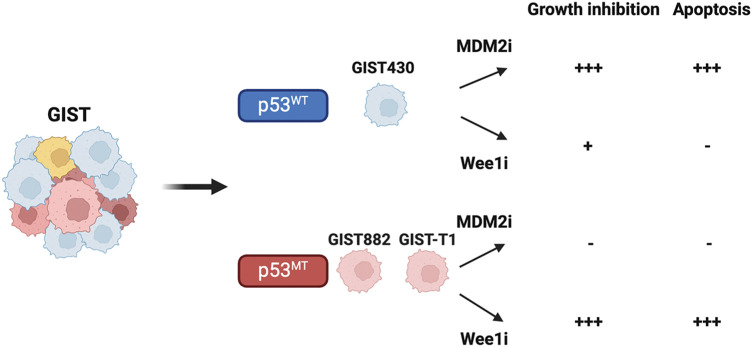
Illustration of cell growth inhibition and apoptosis induced by MDM2 or Wee1 inhibitors (MDM2i or Wee1i), which impair GIST growth depending on the presence of wild-type or mutant p53.-, no effect; +, moderate effect; +++, significant effect. Figure created with Biorender (Toronto, ON, Canada, https://biorender.com)

The study’s findings indicated that HDM201 effectively inhibited cell growth and induced apoptosis in GIST cells harboring wild-type p53. In addition, adavosertib predominantly induced apoptosis in p53 MT GIST cells, but not in p53 WT cells, suggesting a p53-dependent therapeutic response (Fig. 6). These findings are consistent with those of previous studies that reported the antitumor effects of MDM2 inhibitors, including nutlin-3 in osteosarcoma [45], nutlin-3a in leukemia and neuroblastoma [46,47], and HDM201 in melanoma and liver adenocarcinoma [18,48]. Notably, to the best of our knowledge, this is the first *in vivo* study to investigate the therapeutic potential of MDM2 inhibitors in GIST. Furthermore, adavosertib treatment led to a significant increase in caspase-3/7 activity and a higher percentage of cells in the sub-G1 phase, as well as both early and late apoptosis in p53 MT GIST cells (GIST882 and GIST-T1) (Figs. 2 and 3). These findings are consistent with those of previous studies that reported that adavosertib significantly upregulates apoptosis markers, including γ-H2AX and cleaved PARP, in p53 MT cells [14,49], supporting its apoptotic effect in p53-mutant GIST models.

In contrast, the study’s data indicated that adavosertib primarily induced G2/M arrest, with only moderate growth inhibition and no substantial apoptosis, in p53 WT GIST430 cells. This is likely because of the requirement for cell cycle regulation in response to replication stress following G1/S checkpoint deficiency in combination with Wee1 inhibition [30,50]. Following the DNA damage response (DDR), p53 WT cells typically undergo cell cycle arrest at the G1 checkpoint to allow for DNA repair before replication. In contrast, p53 MT cells predominantly rely on the G2 checkpoint for DNA repair [51]. In this study, adavosertib induced S or G2/M phase arrest in p53 MT GIST cells (Fig. 2A,B), consistent with previous reports that elucidated its role in inducing S/G2/M arrest in p53 MT biliary tract cancer, gastric cancer, GIST, and anaplastic thyroid carcinoma (ATC) [14,30,49,52]. Previously, Bukhari et al. (2019) reported that Wee1 inhibition in osteosarcoma cells disrupts mitotic progression, leading to frequent centromere fragmentation and mitotic catastrophe [53]. Similarly, our findings suggest that Wee1 inhibition in p53 MT GIST cells induces DNA content shifts, disrupts cell cycle progression, results in cell cycle arrest at the S or G2/M phases, and ultimately triggers apoptosis. The western blot analysis revealed that adavosertib treatment disrupted cell cycle control in p53 MT GIST cells, leading to increased Wee1 phosphorylation (p-Wee1) and reduced phospho-cdc2 (p-cdc2) expression. The decrease in p-cdc2 suggests the impairment of the cdc2-cyclin complex, which is indicative of DNA damage. Although our results showed an initial upregulation of p-Wee1 following adavosertib treatment, Rajeshkumar et al. reported that this effect was transient [54]. Notably, Wee1 undergoes sequential phosphorylation, leading to its degradation via the ubiquitin-proteasome pathway, which ultimately activates cdc2, promotes mitotic entry, and results in replication stress accumulation [30,54]. Our results suggest that p53-mutant GIST cells may exhibit increased reliance on the G2/M checkpoint, rendering them more vulnerable to Wee1 inhibition. These findings highlight how both KIT and p53 genetic contexts influence drug responsiveness and may inform future combination strategies or biomarker-guided therapeutic approaches.

Although adavosertib efficacy is partially hindered by the presence of functional p53 in p53 WT cells, HDM201 exhibits strong p53-dependent antitumor activity. Our results confirm that HDM201 effectively induces growth inhibition and apoptosis in p53 WT GIST cells by stabilizing p53 and upregulating its downstream targets, while showing limited efficacy in p53 MT cells. These findings align with the *in vitro* results of a previous study that reported that another MDM2 inhibitor, nutlin-3, effectively induced apoptosis in p53 WT GIST cell lines [21]. Unlike tyrosine kinase inhibitors (e.g., imatinib, sunitinib, regorafenib, and ripretinib), which primarily exert cytostatic effects [10], our results showed that HDM201 has the potential to induce apoptosis specifically in p53 WT GIST cells. However, the lack of histopathological examination is a limitation of our current study. In future experiments, we plan to incorporate histopathological evaluations at early time points to verify the on-target effects of our interventions. Specifically, we will increase the number of mice to allow tumor collection at 24 and 48 h following the first and second administration of the inhibitor, enabling us to analyze key protein expression levels, including p53-related proteins (e.g., p21), cell cycle-related proteins (e.g., cdc2), and apoptosis-related proteins (e.g., PARP). Additionally, to assess immune cell infiltration, we will use C57BL/6J (B6) mice for *an in vivo* study, perform H&E staining, and collect tumors for FACS analysis of T cell markers such as CD4 and CD8 [29,55,56]. These analyses will provide a better correlation between on-target effects and our observed outcomes, further elucidating how HDM201 and adavosertib regulate the cell cycle, induce apoptosis, and exert anti-tumor effects based on p53 status.

Despite the promising preclinical data supporting the use of MDM2 inhibitors in GIST, clinical studies targeting p53 in GIST are currently lacking [10,57]. Furthermore, to date, no clinical trials have investigated p53-targeted therapies in patients with GISTs. Given the lack of effective MDM2 inhibitors in clinical practice, further studies are required to explore the therapeutic feasibility of targeting the p53 pathway in GIST. Nevertheless, our results suggest that targeting the p53 pathway may be a potential therapeutic strategy. Importantly, determining the p53 status in patients could facilitate personalized treatment strategies. In low-risk GIST patients, surgical resection is feasible. However, for high-risk and metastatic GIST, we propose a treatment strategy that incorporates p53-targeted therapy into KIT/PDGFRA inhibitor regimens to overcome drug resistance. For high-risk GIST, KIT/PDGFRA inhibitors such as imatinib, sunitinib, regorafenib, and ripretinib could be administered as neoadjuvant therapy to reduce tumor size and stage, followed by surgery and p53-targeted adjuvant therapy to eliminate residual cancer cells and lower recurrence risk. For metastatic GIST, which may not be amenable to surgery, a sequential approach using KIT/PDGFRA inhibitors followed by p53-targeted inhibitors or a combination of both from the outset could be considered [10]. In this context, patients with p53 WT tumors may benefit from an MDM2 inhibitor combined with a KIT/PDGFRA inhibitor, while those with p53 MT tumors might respond more favorably to a Wee1 inhibitor combined with a KIT/PDGFRA inhibitor, thus further enhancing response rates. Specifically, GIST430 cells, which retain wild-type p53 and are imatinib-resistant [58], were selected as the primary model for combination treatment with HDM201 to investigate p53-dependent mechanisms under clinically relevant resistance conditions. In addition, future studies may explore the combination of adavosertib and sunitinib using GIST882 and GIST-T1 cells, both of which harbor mutant p53 and are sensitive to sunitinib [59,60], to evaluate synergistic effects in distinct genetic contexts.

Moreover, to assessment of p53 status in clinical settings remains technically challenging due to both tumor heterogeneity and limitations in current detection methods. While techniques such as RNA sequencing and digital PCR offer high sensitivity for detecting *TP53* mutations [61,62], their clinical applicability may be limited by cost, turnaround time, and the need for high-quality nucleic acid samples. In routine clinical practice, p53 status is often inferred through immunohistochemistry (IHC), which is cost-effective and widely available, but may lack the precision to distinguish between different mutation subtypes or functional consequences. In addition, intra-tumoral heterogeneity can complicate interpretation, especially in the context of sub-clonal mutations or differential expression in metastatic vs. primary lesions. To address these challenges, future approaches may combine multiple modalities, such as next-generation sequencing (NGS) panels with digital PCR validation and IHC for functional protein assessment to achieve a more comprehensive and clinically actionable evaluation of p53 status. The development of liquid biopsy-based assays (such as circulating tumor DNA) may also offer a non-invasive method to monitor *TP53* mutations over time and across tumor sites, potentially improving real-time therapeutic decision-making [63,64].

Regarding the specific *TP53* mutations in the GIST882 and GIST-T1 cell lines, we mentioned that GIST882 carries a *TP53* exon 1 deletion, and GIST-T1 harbors a larger homozygous deletion spanning exons 2–7 previously [21]. Both mutations lead to loss-of-function alterations, resulting in a p53-null phenotype with complete loss of p53 transcriptional activity (Fig. 4), rather than missense or nonsense mutations that might confer dominant-negative or gain-of-function effects. We acknowledge that the current study does not deeply explore the functional consequences of different *TP53* mutation subtypes on the efficacy of adavosertib. This represents a limitation, as varying *TP53* mutation types could influence therapeutic responses differently, particularly in cases with retained or neomorphic p53 function. Concerning *in vivo* validation, our study included *in vivo* testing with adavosertib as a single agent therapy in the GIST882 model, which demonstrated efficacy in p53-null cell lines. However, we did not perform *in vitro* and *in vivo* experiments evaluating combination therapies. This is a notable limitation, as combining adavosertib with other targeted therapies (such as KIT inhibitors) could potentially enhance efficacy or overcome resistance mechanisms in GIST, particularly in the context of *TP53* mutations. Future studies should prioritize *in vivo* validation of combination therapies using GIST models with diverse *TP53* mutation profiles to better elucidate synergistic effects and differential sensitivities.

This study’s findings highlight the distinct therapeutic responses to HDM201 and adavosertib according to p53 status, underscoring the significance of p53 as a biomarker for guiding treatment selection in GIST. However, because this combination strategy has yet to be evaluated in a preclinical setting, further research is needed to establish a foundation for clinical trials. Moreover, to further overcome resistance, combination therapy with TKIs and other agents may improve treatment efficacy. Resistance to HDM201 may arise from MDM2 amplification or overexpression, which could enhance p53 degradation in *TP53* wild-type cells [65,66], though this is less relevant in p53-null models like GIST882 and GIST-T1. Additionally, mutations in downstream p53 signaling pathways (such as p21 or BAX) could diminish HDM201 efficacy. For adavosertib, resistance may stem from compensatory activation of alternative DNA damage response pathways, such as ATR/Chk1 signaling, or upregulation of other G2/M checkpoint regulators [67,68]. In GIST, KIT secondary mutations could also contribute to resistance by sustaining oncogenic signaling despite Wee1 inhibition. HDM201 and adavosertib could complement existing GIST therapies, particularly for patients with imatinib-resistant tumors driven by KIT secondary mutations or *TP53* alterations. HDM201 use in *TP53* wild-type GISTs, where MDM2 inhibition can restore p53 function, potentially synergizing with tyrosine kinase inhibitors (TKIs) like imatinib or sunitinib. Adavosertib, effective in p53-null models, could be integrated as a second or third-line therapy for imatinib-refractory cases, particularly in combination with TKIs to target both KIT-driven proliferation and DNA damage response vulnerabilities. In addition, novel targets are under investigation example, CDK1 has been identified as a key regulator of GIST growth and proliferation, suggesting that CDK1 inhibitors could be potential therapeutic agents [69]. Immunotherapeutic strategies, such as combining immune checkpoint inhibitors with anti-angiogenic agents, have shown promise in GIST treatment [70]. Furthermore, many new drugs are currently in clinical trials for advanced GISTs, with some already entering phase III trials, including targeting PDGFRα/β, VEGFR, or GTPase [71]. These agents show promising efficacy and safety profiles, but more clinical data are needed to fully confirm their benefits.

## 5 Conclusions

This study revealed that HDM201 exerts potent p53-dependent antitumor effects by inducing apoptosis in p53 WT GIST cells. In contrast, adavosertib promoted G2/M arrest and apoptosis primarily in p53 MT GIST cells. *In vivo* studies further validated the efficacy of HDM201 in reducing the tumor burden in p53 WT GIST430 xenografts, whereas adavosertib showed modest effects in p53 MT GIST882 models. These findings provide a strong rationale for the development of tailored therapeutic strategies targeting the p53 pathway in GIST.

## Supplementary Materials



## Data Availability

The data in this study are available upon email request to the corresponding author, Chiao-En Wu.

## Supplementary Materials

The supplementary material is available online at https://www.techscience.com/doi/10.32604/or.2025.066672/s1.
