# Supplementary material for: Efficacy of Wee1 G2 Checkpoint Kinase and Mouse Double Minute 2 Homolog Inhibitors in Gastrointestinal Stromal Tumors Determined by p53 Status
Source: Oncol Res. 2025 Oct 22;33(11):3429–46. doi: 10.32604/or.2025.066672 (PMC12573211; doi:10.32604/or.2025.066672)
Supplement: Supplementary file 1 [file OncolRes-33-66672-s001.docx]

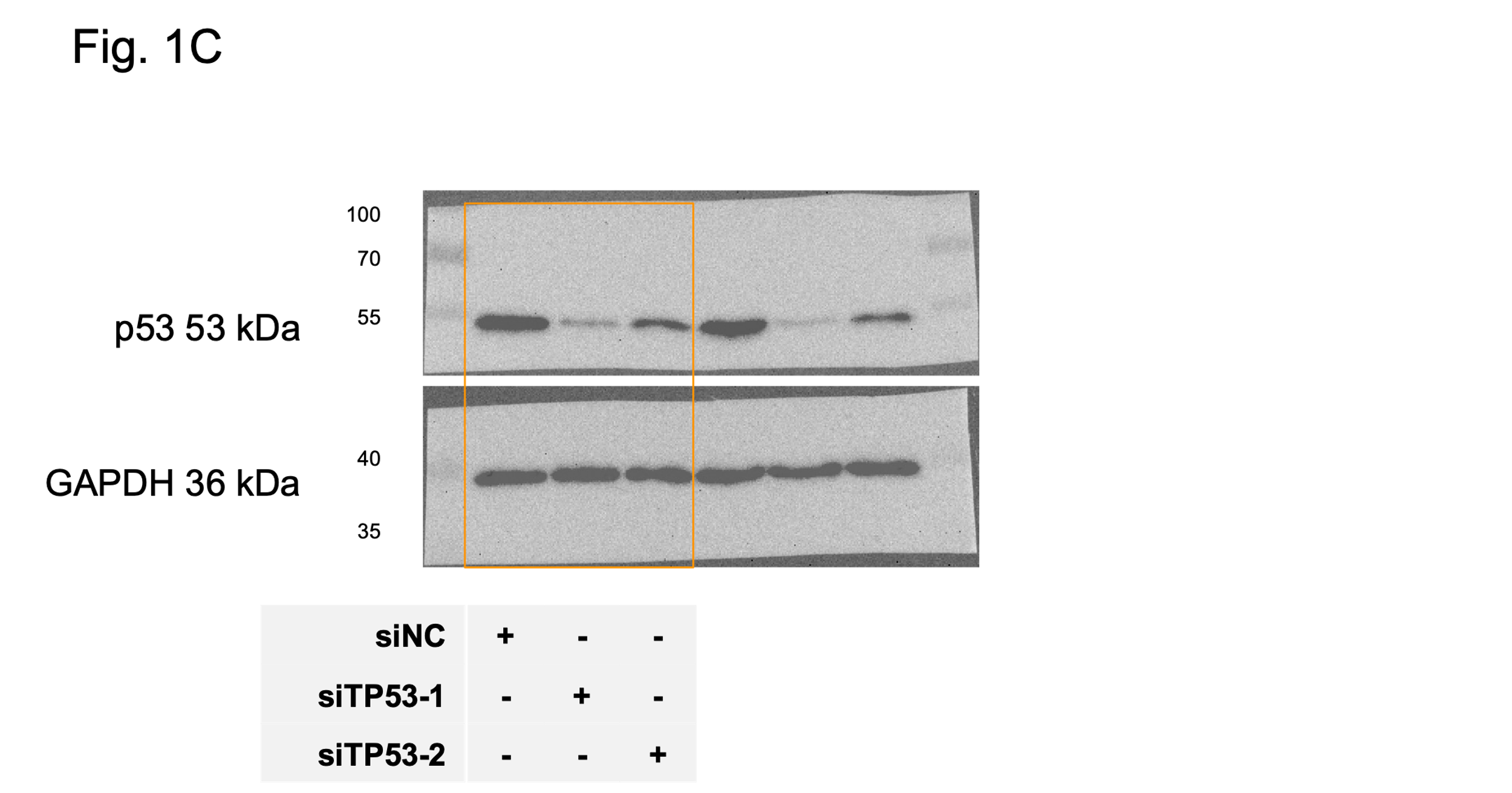


**Figure S1: Uncropped western blot of Fig. 1C**

**
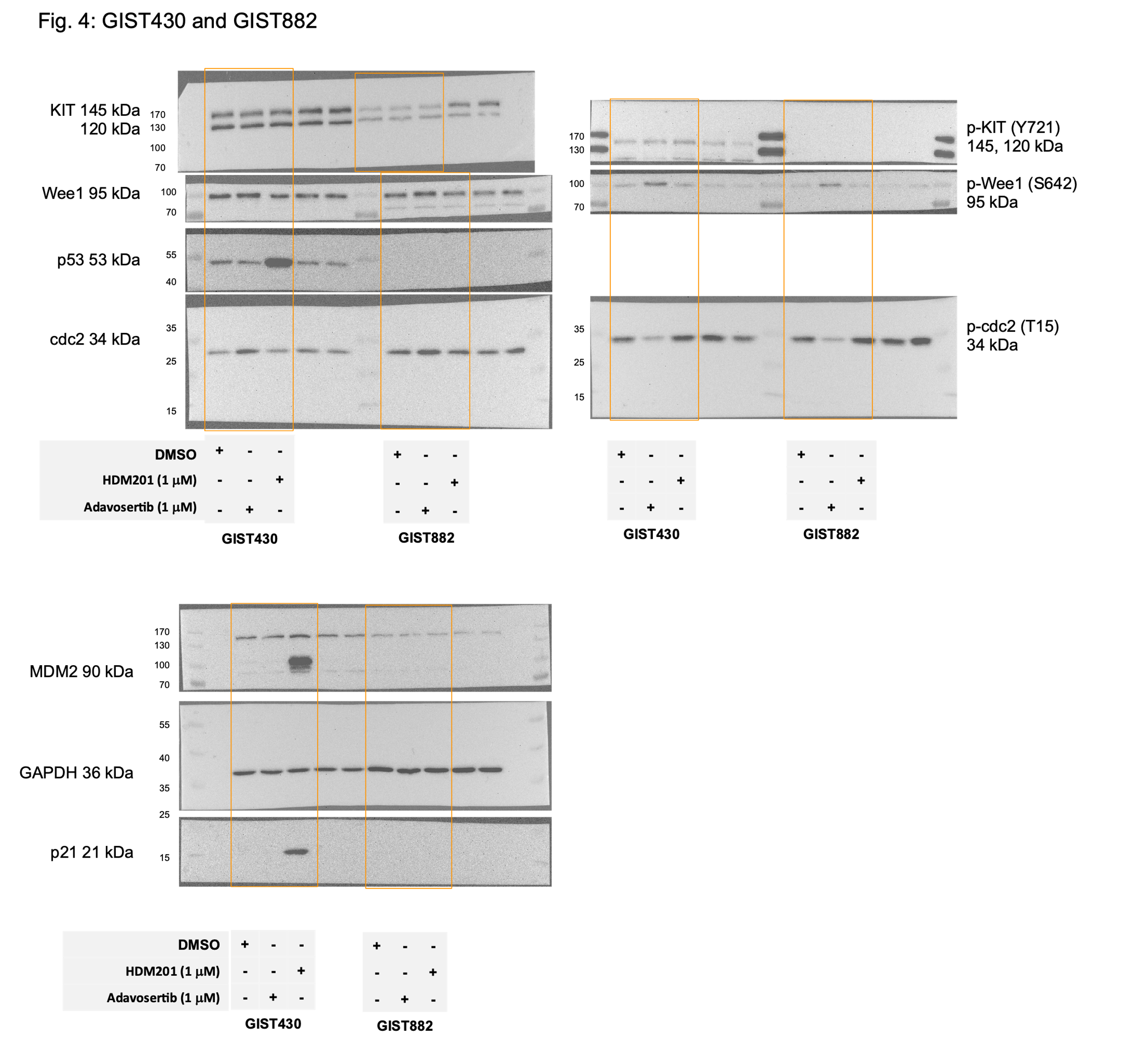
**

**Figure S2: Uncropped western blot of Fig. 4 (GIST430 and GIST882)**

**
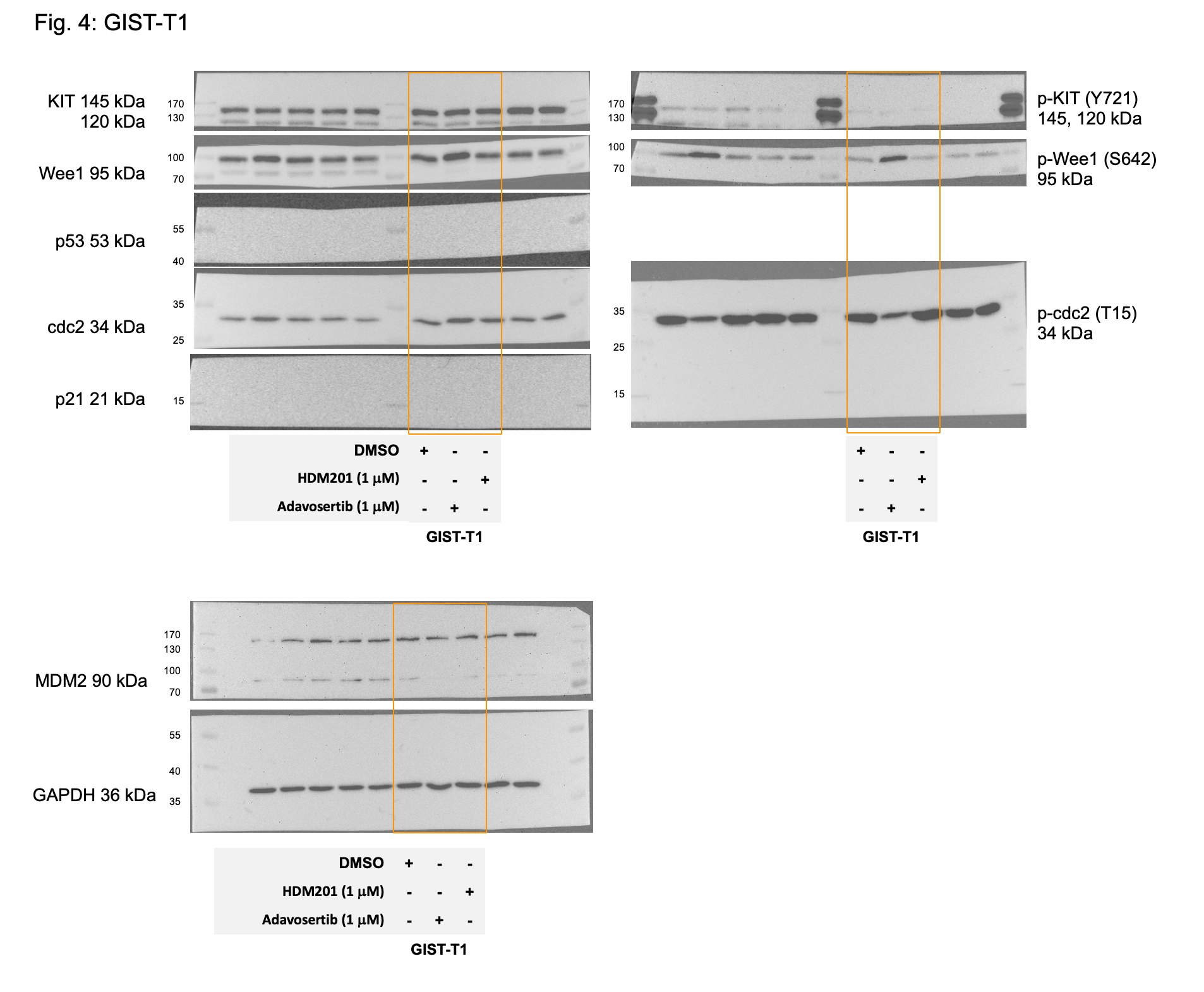
**

**Figure S3: Uncropped western blot of Fig. 4 (GIST-T1)**
